# Traditional Chinese medicine in the era of immune checkpoint inhibitor: theory, development, and future directions

**DOI:** 10.1186/s13020-023-00751-7

**Published:** 2023-05-20

**Authors:** Yi-xuan Yu, Shuo Wang, Zhe-ning Liu, Xu Zhang, Zi-xin Hu, Hui-jing Dong, Xing-yu Lu, Jia-bin Zheng, Hui-juan Cui

**Affiliations:** 1 Graduate School, Beijing University of Chinese Medicine, Beijing, 100029 China; 2 Oncology Department of Integrative Medicine, China-Japan Friendship Hospital, No.2 Yinghua East Road, Chaoyang District, Beijing, 100029 China

**Keywords:** Traditional Chinese Medicine, Immune checkpoint inhibitor, Immunotherapy, Tumor microenvironment

## Abstract

Immune checkpoint inhibitors (ICIs) have revolutionized cancer management and have been widely applied; however, they still have some limitations in terms of efficacy and toxicity. There are multiple treatment regimens in Traditional Chinese Medicine (TCM) that play active roles in combination with Western medicine in the field of oncology treatment. TCM with ICIs works by regulating the tumor microenvironment and modulating gut microbiota. Through multiple targets and multiple means, TCM enhances the efficacy of ICIs, reverses resistance, and effectively prevents and treats ICI-related adverse events based on basic and clinical studies. However, there have been few conclusions on this topic. This review summarizes the development of TCM in cancer treatment, the mechanisms underlying the combination of TCM and ICIs, existing studies, ongoing trials, and prospects for future development.

## Introduction

Cancer is one of the leading causes of death globally, with almost 10 million cancer-related deaths reported in 2020 [1]. Recently, immunotherapies, represented by immune checkpoint inhibitors (ICIs) have revolutionized cancer management. Immune checkpoints can be expressed by tumor cells to escape immunosurveillance [2, 3]. The most common immune checkpoints are cytotoxic T lymphocyte-associated protein 4 (CTLA-4) and programmed cell death 1 (PD-1) [4]. ICIs are monoclonal antibodies targeting immune checkpoint molecules that can restore the body’s antitumor immune response and promote T cell-mediated clearance of tumor cells by blocking the inhibitory signaling pathways of T cells. ICIs have become the first-line treatment for a variety of solid and liquid tumors. Despite the development of ICIs treatment, numerous problems remain, including uncertain efficacy, immune checkpoint inhibitor-related adverse events (irAEs), and drug resistance.

China is a large country with high cancer burden. The integration of Traditional Chinese Medicine (TCM) and Western medicine is a special therapy that is implemented in more than 70% of cancer patients in China [5]. TCM treatments include the use of Chinese medicine monomers, extracts, traditional compound prescriptions (a prescription consisting of two or more TCM herbs), Chinese medicine patents (a TCM product composed of TCM herbs and processed according to the prescribed prescription and preparation process [6]), acupuncture, exercise (slow, gentle, and symmetrical movements represented by Tai Chi, Baduanjin, Yijinjing, and Wuqinxi [7]), and moxibustion (burning of the herb moxa over acupuncture points [8]) [9]. Previous studies have suggested that TCM can suppress angiogenesis, growth, and metastasis of tumor cells, and prompt their apoptosis [10, 11], which has achieved excellent curative outcomes in the treatment of cancer.

*Yin* and *Yang*, a concept derived from ancient Chinese philosophy, describe how opposite or contrary forces may be complementary, interconnected, and interdependent. *Yin* refers to things, nature, or body functions that are cold, downward, inert, dim, internal, material, declining, and inhibiting [12, 13]. *Yang* is the opposite of *Yin*. Hence, the concepts of *Yin* (negative regulation) and *Yang* (positive regulation) are often associated with immune function [14] and maintenance of the immune balance [15]. Studies have increasingly demonstrated the use of TCM for combining ICIs through immunomodulation, enhancing the efficacy of ICIs, reducing the incidence of irAEs, and treating irAEs [16, 17]. Nevertheless, the combination of TCM and ICIs has received limited attention, and its mechanism and efficacy remain unclear. Herein, we aimed to provide an overview of the combination of TCM and ICIs including potential mechanisms, current studies, and our perspective on the future.

## Development of TCM in oncology treatment

The connotation of the tumor was first discussed in *The Yellow Emperor’s Inner Canon*, which was a classical work of TCM more than 2000 years ago [18]. This masterpiece has proposed various classifications and the corresponding names of tumors including *“Shijia”*, *“Changqin”*, *“Jinliu”*, *“Jiju”*, *“Yege”*, etc. Through long-term clinical practice, TCM has summarized the etiology of tumors as external causes of environmental factors, for instance, environmental pollution, and six pathogenic factors from the environment, namely, wind, cold, summer-heat, dampness, dryness, and fire; internal injuries caused by emotional factors (seven emotions consisting of joy, anger, worry, thought, grief, fear, and surprise), overstraining, and improper diet are also discussed.

TCM has a unique understanding of the pathogenesis and development of oncology, including dysregulation of *Yin* and *Yang*, disturbances in circulation of the two basic substances of *Qi* and *Xue*, and disorders of *Zang* and *Fu* representing the internal organs and their function. Hence, TCM has proposed that the overall treatment principles are strengthening body resistance and eliminating evil, which is regulating *Yin* and *Yang*, *Qi* and *Xue*, and *Zang* and *Fu*.

In the past 70 years, integrated TCM and Western medicine have been the most distinctive methods for the treatment of cancers in China. The main treatment methods include TCM combined with radiotherapy [19], chemotherapy, targeted therapy [20], immunotherapy, and maintenance therapy for end-stage patients. During the different phases of cancer, TCM has shown a significant antitumor effect, reversing drug resistance, alleviating clinical symptoms, decreasing treatment-related adverse events, improving quality of life, and extending overall survival (including precancerous lesions [21], neoadjuvant treatment [22], adjuvant treatment [23], supportive care [24], and prevention of recurrence [25]).

Various TCM methods have been used to develop generalizable treatment regimens. As early as 1997, China’s State Food and Drug Administration had already approved the use of Kanglaite (extracted from the Chinese herb, Coicis semen yokuinin) for hepatic cell carcinoma (HCC) (odds ratio [OR] = 2.57, 95% confidence interval [CI] 2.10–3.16, *P* < 0.00001) [26] and non-small cell lung cancer (NSCLC) (relative risk [RR] = 1.45, 95% CI 1.31–1.60, *P* < 0.00001) [27], which would increase the objective response rate (ORR) combined with chemotherapy [28]. Aside from the development of TCM injections and oral Chinese patent medicine [29], new drugs were discovered from TCM, such as Haier granules [30] and Icaritin [31], which were recommended by the Chinese Society of Clinical Oncology clinical guidelines as standard drugs.

Furthermore, basic research has been conducted on the complex and systematic anticancer mechanisms of TCM at the molecular and cellular levels, particularly in immunomodulation [16]. *Ginseng* [32, 33] and *Astragalus* [34] have been shown to affect both innate and adaptive immunity. The mechanisms of TCM on DNA methylation, histone modification, and regulation of noncoding RNAs are being explored [35, 36]. Artificial intelligence-assisted TCM promises to become a new growth point [37]. Using digital data, more solid evidence for TCM will be provided.

In recent years, TCM guidelines and international cooperation have been developed in the field of cancer [19]. Increasing international communication and standardizing TCM has contributed to its development. Consequently, TCM deserves more attention in the immuno-oncology era.

## Mechanisms of TCM impacts ICI therapy and irAEs

### Regulation of tumor microenvironment: stromal cells, immune cells, and hypoxia

The tumor microenvironment (TME) is the environment where tumors originate and develop. Growing evidence suggests that TCM can influence stromal components, immune cells, and metabolic status to reverse the resistance of ICIs, enhance clinical efficacy, and prevent and reduce the severity of irAEs [38, 39].

TCM can inhibit angiogenesis by regulating vascular endothelial growth factor (VEGF) expression, reducing the activity of angiogenic factor receptors, or inhibiting endothelial cell proliferation [40]. Various herbs, including *Astragalus Membranaceus* and *Curcuma Wenyujin* [41] promote vascular normalization in tumor-derived endothelial cells of HCC. These effects prevent abnormal vasculature and high interstitial pressure within the tumor, resulting in a high level of immune cell infiltration and ICIs penetration [42].

TCM also plays a crucial role in regulating immune cell infiltration and activity in the TME, including T cells, natural killer cells (NKs), regulatory cells (Tregs), tumor-associated macrophages (TAMs), and myeloid-derived suppressor cells (MDSCs). The sensitization effect of TCM is enhanced by higher infiltration of CD8 + and CD4 + T cells, while immunity suppressors and TAMs are decreased [43]. TCM balances immune responses and tolerances in terms of treating and preventing irAEs. Despite the lack of a complete understanding of how irAEs function, increasing research suggests that TCM can be used to treat irAEs by enhancing immunosuppressive cells [44, 45]. TCM appears to have a double-edged sword for sensitizing efficacy and for treating irAEs. Consistent with this, TCM exerts its effects both in the treatment of infection [46] and autoimmune diseases [47, 48].

Hypoxia is another characteristic of TME that hinders ICI efficacy [49]. TCM alleviates tumor hypoxia in the TME by suppressing hypoxia-inducible factor 1α (HIF-1α) [50] and lactate [51]. For example, *Rhodiola crenulate*, a well-known Chinese herb, can resist hypoxia through increased VEGF, poor perfusion, and inhibition of hypoxia-inducible transcription factor signaling [52–54].

### Modulation of gut microbiota

First, the gut microbiota influences systemic immune function and antitumor immunity. Several studies have demonstrated that the diversity of gut microbiota affects the response of ICIs [55]. A variety of species, including *Bifidobacterium* [56], *Akkermansia*, and *Alistipes* [57] can boost ICI responses. The gut microbiota also affects the incidence and severity of irAEs [58, 59]. Patients with melanoma who are treated with ICIs and rich in *Bacteroides dorei* are more likely to develop irAEs [60].

The interaction between the gut microbiota and TCM is a key mechanism for the combination of TCM and ICIs [61]. On the one hand, gut microbiota transforms TCM compounds into active chemicals. On the other hand, TCM regulates the gut microbiota to increase immunity, promote the effects induced by ICIs, balance the immune response, and treat irAEs [62]. Basic studies, such as those on the TCM Shaoyao Ruangan mixture and berberine suggest that TCM leads to an increase in the diversity of gut microbiota, wherein a higher abundance of *Bifidobacterial* [63], *Lactobacillus* [64], *Firmicutes*, and a lower abundance of Proteobacteria [65] is noted, which are associated with good ICI efficacy, fewer irAEs, and milder symptoms.

### Other possible mechanisms

PD-L1 is expressed in tumor cells, and the combination of PD-L1 and PD-1 leads to tumor immune escape. As a natural immune checkpoint inhibitor, TCM can suppress PD-L1, which inhibits PD-1/PD-L1 interaction. As a result of the deubiquitinating activity of berberine’s, PD-L1 is ubiquitinated and degraded, inhibiting the PD-1/PD-L1 axis [66]. Bu-Fei Decoction was found to suppress the expression of PD-L1 by Pang et al. [67]. Zhang et al. [68] demonstrated that the TCM compound prescription CFF-1 suppressed PD-L1 expression in prostate cancer cells in a time- and dose-dependent manner. However, high expression of PD-L1 is considered a prerequisite for PD-1/PD-L1 immunotherapy [69], and whether the TCM potentiates ICIs through this mechanism is unclear.

The bidirectional function of cytokines has also been demonstrated during ICI treatment. Cytokines such as interleukin-2 (IL-2) and IL-12 have previously been introduced to increase intertumoral lymphocyte infiltration and anti-tumor immunity [70, 71], but their production in response to ICIs can increase irAEs. Cytokine inhibitors targeting tumor necrosis factor-α (TNF-α), IL-6, and IL-17A have been increasingly used to treat irAEs [72]. Some TCM treatments can enhance cytokine production, whereas others can prevent these cytokines from increasing to treat irAEs [73, 74]. Diammonium glycyrrhizinate [75], extracted and purified from liquorices, improves the production of IL-6 and IL-7, protecting the liver from injury. However, TCM can inhibit cytokine storm including IL-6, and TNF-α [76].

**Fig. 1:**
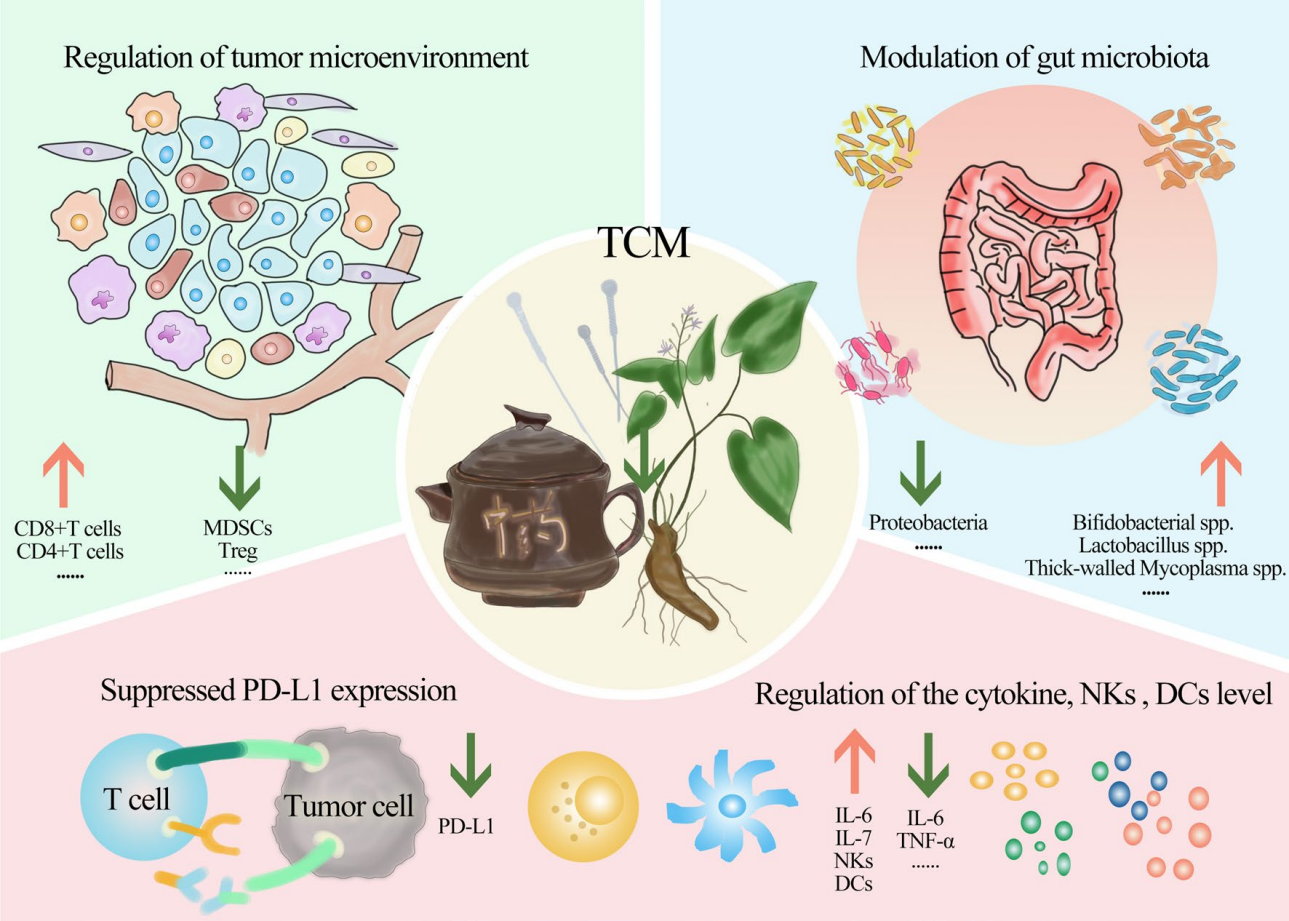
Mechanisms of TCM impacts the ICIs therapy and irAEs

There is also promising evidence that TCM can increase NKs infiltration in lymph nodes and immune organs, enhance dendritic cell (DC) activity, and improve the efficacy of ICIs [77, 78] Fig. 1.

## TCM enhances the efficacy of ICIs and reverses drug resistance

Clinical and experimental studies have shown that TCM and ICIs are more effective in combination. The synergistic effect of TCM has been demonstrated in mouse models of colon carcinoma [79–87], lung cancer [88, 89], breast cancer [84, 87], melanoma [91–94], ovarian cancer [95], HCC [96], and acute T lymphoblastic leukemia [97] (Table 1).

**Table 1:** The basic research on TCM enhances the efficacy of ICIs

Cancers (cells)	Type	TCM	Formulation	ICIs	Upregulate	Downregulate	References
Mechanism: regulation of tumor microenvironment							
Colon carcinoma (CT26)	In vivo and in vitro	*Andrographis paniculata*	Monomer	Anti-PD-1	CD4 + and CD8 + T cells, IFN-γ, and granzyme B +	COX2 and PGE2	Liu W et al. [79]
Colon carcinoma (MC38)	In vivo and in vitro	*Sanguisorbae Radix*	Extract	Anti-PD-1	CD8 + T cells	NA	Lee EJ et al. [86]
Colon carcinoma (CMT93 and HCT116)	In vivo and in vitro	Pien-Tze-Huang	Compound prescription	Anti-PD-1 and anti-PD-L1	CD3 + and CD8 + T cells, IFN-γ, and granzyme B +	p-STAT3, IRF1, and PD-L1 expression	Chen Q et al. [88]
Colon carcinoma (CT26) and breast cancer (4T1)	In vivo	Ginseng-derived nanoparticles	Monomer	Anti-PD-1	CD4 + and CD8 + T cells, CCL5, CXCL9, granzyme B + , IFN-γ, TNF-α, and IL-2	M2/M1 ratio	Han X et al. [84]
Colon carcinoma (MC38)	In vivo	Dahuang Fuzi Baijiang decoction	Compound prescription	Anti-PD-1	CD8 + T and PD-1^int^ T cells	PD-1^hi^Tim3 + Tex	Xu Y et al. [83]
Colon carcinoma (MC38)	In vivo	Atractylenolide I	Monomer	Anti-PD-1	CD8 + T cells	NA	Xu H et al. [85]
Breast cancer (4T1 or MDA-MB-231)	In vivo	Salvianolic acid B	Monomer	Anti-PD-L1	CD8 + T cells, granzyme B + , IFN-γ	PD-1 expression	Qian C et al. [91]
Melanoma (B16F10) and colon carcinoma (MC38) and	In vivo and in vitro	*Icaritin*	Monomer	Anti-PD-1 and anti-CTLA-4	CD8 + T cells	PD-L1 expression on MDSCs and neutrophils	Hao H et al. [93]
Melanoma (B16-F10)	In vivo and in vitro	*Ailanthone*	Monomer	Anti-PD-L1	CD4 + and CD8 + T cells, granzyme B + , IFN-γ, M1	Tregs, M2, MDSCs, and PD-L1 expression	Yu P et al. [94]
Lung cancer (A549 or lewis lung carcinoma)	In vivo and in vitro	Cryptotanshinone	Monomer	Anti-PD-L1	CD4 + and CD8 + T cells, CXCL9, CXCL11, granzyme B + , IFN-γ, and perforin	DCs (CD11c + /CD45 +)	Liu S et al. [90]
Ovarian cancer (JHOC-5), colon cancer (MC38 or CT26)	In vivo and in vitro	Curcumin	Monomer	Anti-PD-1 and anti-PD-L1	CD8 + T cells, IFN-γ, DCs in the dLNs and spleens	STAT3 and IL-6	Hayakawa T et al. [95]
Hepatocellular carcinoma (Hepa1-6)	In vivo	Bufalin	Monomer	Anti-PD-1	CD4 + and CD8 + T cells, macrophage, IFN-γ, TNF-α, and IL-10	M2 phenotype, p50 NF-κB, and TGF-β	Yu Z et al. [96]
Mechanism: modulation of gut microbiota							
Colon carcinoma (CT26)	In vivo	Gegen Qinlian decoction	Compound prescription	Anti-PD-1	*s__Bacteroides_acidifaciens*, *s__uncultured_organism_g__norank_f__Bacteroidales_S24-7_group*, CD8 + T cells, IL-1, and IFN-γ	CD4 + T cells	Lv J et al. [81]
Colon carcinoma (MC38)	In vivo	Jujube powder	TCM	Anti-PD-L1	Diversity index of gut microbiota, and *Lachnospiraceae*	*Prevotellaceae*	Wang L et al. [80]
Lung cancer (lewis lung cancer) and melanoma (B16-F10)	In vivo	Ginseng polysaccharides	Monomer	Anti-PD-1	Diversity index of gut microbiota, and *Bacteroides* (*B. vulgatus* and *P. distasonis*), CD8 + T cells, granzyme B + , IFN-γ, TNF-α, and SCFAs abundance	Treg cells, IDO activity, kynurenine/tryptophan ratio	Huang J et al. [89]
Mechanism: others							
Melanoma (B16)	In vivo and in vitro	Juzentaihoto	Compound prescription	Anti-PD-1	IL-12, IFN-γ, and NKs activity	NA	Ishikawa S et al. [93]
Colon carcinoma (CT26)	In vivo and in vitro	Jiedu Sangen decoction	Compound prescription	Anti-PD-L1	E-cadherin	N-cadherin, β-actin, Slug, Snail, Vimentin, PI3K, P-AKT, and AKT	Shan F et al. [82]
Melanoma (B16F10)	In vivo	Astragalus membranaceus polysaccharides	Monomer	Anti-PD-L1	NKs, IFN-γ, CD4 + and CD8 + T cells in mesenteric lymph nodes and lung	B16 cells	Hwang J et al. [92]
Acute T lymphoblastic leukemia (Jurkat cells)	In vitro	YIV-906 (inspired by Huang Qin Tang)	Compound prescription	Anti-PD-1	Nuclear factor of activated T cells activity	NA	Lam W et al. [97]

*TCM* traditional Chinese medicine, *ICIs* immune checkpoint inhibitors, *PD-1* programmed death-1, *IFN-γ* interferon-γ, *COX2* cyclooxygenase-2, *PGE2* prostaglandin E2, *NA* not available, *PD-L1* programmed cell death-ligand 1, *p-STAT3* phosphorylated signal transducer and activator of transcription 3, *IRF1* interferon regulatory factor 1,*CCL5* chemokine (C-C motif) ligand 5, *CXCL9* chemokine (C-X-C motif) ligand 9, *CXCL11* chemokine (C-X-C motif) ligand 11, *TNF-α* tumor necrosis factor-α, *IL-2* interleukin-2, *M2* macrophages 2, *M1* macrophages 1, PD-1int T cells intermediate PD-1 expression, *PD-1hiTim3* + Tex T-cell immunoglobulin domain and mucin domain 3 + subset with intermediate expression of PD-1, *MDSCs* myeloid-derived suppressor cells, *Tregs* regulatory cells, *DCs* dendritic cells, *dLNs* draining lymph nodes, *IL-6* interleukin-6, *IL-10* interleukin-10, *p50 NF-κB* nuclear factor kappa-B1, *TGF-β* tumor necrosis factor-β, *IL-1* interleukin-1, *SCFAs* short chain fatty acids, *IDO* indole-3-pyruvate, *IL-12* interleukin-12, *NKs* natural killer cells, *PI3K* phosphoinositide-3 kinase, *P-AKT* phosphorylated protein kinase B

By modulating the TME, Andrographolide, the main bioactive component of TCM Andrographis paniculate, inhibits tumor growth (tumor weight of 1.57 g from 2.53 g) and induces apoptosis with an increase in infiltration and function of CD4 + and CD8 + T lymphocytes with increased tumor suppression cytokines, including interferon-γ (IFN-γ) (combination group vs. anti-PD-1 group:16.5 ± 1.7% vs. 10 ± 1.6%, *P* < 0.05), perforin, granzyme B, recombinant factor related apoptosis ligand (FasL), and TNF-α in colon cancer mouse models combined with anti-PD-1 [79]. By enhancing the infiltration of CD8 + T and CD4 + T cells in the TME, Cryptotanshinone accelerates anti-PD-L1 activity in a lung cancer model with high expression of chemokine (C-X-C motif) ligand 9 (CXCL9), CXCL11, and granzyme B + [89]. One Ginseng-derived nanoparticle reprograms TAMs to increase chemokine (C-C motif) ligand 5 (CCL5) and CXCL9 secretion to recruit CD8 + T cells, which synergizes with anti-PD-1 [84]. *Sanguisorbae Radix* efficiently enhances tumor-infiltrating CD8 + T cell activation by blocking the PD-1/PD-L1 interaction in colorectal cancer (CRC), promoting the efficacy of anti-PD-1 [86]. Salviaric acid B, another active ingredient in salvia, potentiates CD8 + T cell infiltration in the TME along with endothelial protection resulting in the normalization of vascular function and inducing a positive efficacy of anti-PD-L1 in breast carcinoma models [87]. Of note, *Ailanthone* plays a synergistic effect by reducing the infiltration of immunosuppressive Tregs (combination group vs. vehicle + anti-PD-L1:5.13 vs. 18) in melanoma model [94]. In the presentation of tumor antigens, curcumin administration restored the T cell stimulatory activity of murine DCs in murine tumor models, leading to synergistic antitumor activity with anti-PD-1/PD-L1 [95].

TCM also enhances the effects of ICIs in regulating the gut microbiota. In a lung cancer model, the combination therapy of oral ginseng polysaccharides and anti-PD-1 sensitizes the antitumor effect by increasing *Muribaculum* abundance in the combination group compared to the anti-PD-1 alone group [88]. Oral jujube powder [80] elevates the alpha diversity index of gut microbiota and the abundance of *Lachnospiraceae*, leading to promising efficacy of anti-PD-L1 against the colon tumor model. The classic TCM formulation Gegen Qinlian Decoction [81] has been shown to significantly improve antitumor efficacy by continuously enriching *Bacteroides acidifaciens* and, *Peptococcaceae* over time.

Based on other possible mechanisms, Hwang et al. [91] found that intranasal treatment with a membranaceus polysaccharide activated DCs in the mesenteric lymph nodes (mLNs), and stimulated NKs and T cells in the mLNs, and enhance anti-PD-L1 activity in B16 melanoma cells. Ishikawa et al. [93] also indicated that the TCM compound prescription Juzentaihoto increases IL-2, IFN-γ, and NKs activity, leading to a promising effect of anti-PD-1.

Studies of patients treated with TCM and ICIs, particularly in English, have reported limited results. In a retrospective study conducted by Tsao et al. [98], neutrophil-to-lymphocyte ratios were decreased by astragalus polysaccharide injections, ICIs, and chemotherapy (treatment group vs. control group: 0.11 vs. 0.52, *P* = 0.003). The overall survival (OS) was prolonged, but not statistically significant (treatment group vs. control group: 26.1 months vs. 25.4 months, *P* = 0.76). The ORR for Xiaoyan decoction was higher than that for nivolumab alone in a randomized clinical trial [99] for advanced NSCLC (treatment group vs. control group: 57.14% vs. 28.00%, *P* < 0.05).

Currently, 15 clinical trials are registered with ClinicalTrials.gov and the Chinese Clinical Trial Registry (ChiCTR) (Table 2). In those clinical trials, TCM compounds (11, 73.3%) were favored over monomers and other treatments. These compounds include Xianglian Wan (ChiCTR1900026300), Yiqijiedu compound (ChiCTR2000036977, ChiCTR2100041920), Bushen Tiaoyuan Recipe (ChiCTR2000032287), Shenlingbaizhu Powder (ChiCTR2200061279), HuGuXiaoJiTang (NCT05378334), Fuzheng Kangai Granule (ChiCTR2200055453), Gegen Qinlian Tablets (ChiCTR2100051747), Fuzi Lizhong Pill (ChiCTR2200058126), and Wenyang Tongluo Recipe (ChiCTR2200055330), which include ancient classic prescriptions as well as self-prescribed medications. Combination regimens typically used anti-PD-1 rather than anti-PD-L1 or anti-CTLA-4 (12, 80.0%). The cancer type with the most ongoing trials was lung cancer (10, 66.7%). Randomized and parallel clinical trials have been widely conducted to test whether TCM can enhance the efficacy of ICIs, which is associated with robust evidence.

**Table 2:** Ongoing clinical trials on enhancing the effect of ICIs by TCM

Year-author	Registry number	Cancer type	TCM	Formulation	Intervention	Controlled	Sample size	Primary outcomes
RCT								
2019-Zhong Y	ChiCTR1900026300	Malignant tumors	Xianglian Wan	Compound prescription	ICIs + Xianglian Wan	ICIs + Xianglian Wan placebo	44/44	PFS and intestinal flora
2020-Li X	ChiCTR2000040911	Lung cancer	Astragalus Polysaccharide	Monomer	Anti-PD-1(Carrelizumab) + apatinib mesylate + Astragalus polysaccharide injection	Anti-PD-1(carrelizumab) + apatinib mesylate	30/30	PFS
2020-Yang J	ChiCTR2000036977	NSCLC	Yiqijiedu compound	Compound prescription	Anti-PD-1(Pembrolizumab) + Yijiedufang Compound	Anti-PD-1(pembrolizumab)	30/30	PFS and MST
2021-Yang J	ChiCTR2100041920	NSCLC	Yiqijiedu compound	Compound prescription	Anti-PD-1 + Yijiedufang Compound	Anti-PD-1 + placebo	40/40	PFS and OS
2021-Tian W	ChiCTR2100045870	HCC	Huaier Granules	Chinese patent medicine	Anti-PD-L1(atelizumab) + bevacizumab + Huaier Granules	Anti-PD-L1(atelizumab) + bevacizumab	90/90	Tumor size, AFP, and safety
2021-Yan D	ChiCTR2100051276	NSCLC	Baicalin combination	Monomer	Anti-PD-1 + baicalin combination	Anti-PD-1	76/76	Tumor size
2022-Wu W	ChiCTR2200055453	NSCLC	Fuzheng Kangai Granule	Compound prescription	Anti-PD-1 + Fuzheng Kangai Granule	Anti-PD-1 + placebo	30/30	PFS
2022-Feng C	ChiCTR2200061279	NSCLC	Shenlingbaizhu Powder	Compound prescription	Anti-PD-1 + Shenlingbaizhu Powder	Anti-PD-1	88/88	PFS
2022-Zhang H	NCT05378334	NSCLC	HuGuXiaoJiTang	Compound prescription	Anti-PD-1 + chemotherapy + HGXJT	Anti-PD-1 + chemotherapy	41/41	DCR
Single-arm study								
2021-Li P	ChiCTR2100049159	HCC	TCM	Compound prescription	Anti-PD-1(carrelizumab) + TCM	NA	50	PFS
2021-Cui H	ChiCTR2100051747	LUSC	GegenQinlian Tablets	Compound prescription	Anti-PD-1 + GegenQinlian Tablets	NA	27	ORR and DCR
2021-Cao Y	ChiCTR2100046431	Lung cancer	Gensing Polysaccharides	Compound prescription	ICIs + Gensing Polysaccharides	NA	45	ORR and DCR
2022-Wang G	ChiCTR2200058126	Pancreatic cancer	Fuzi Lizhong Pill	Compound prescription	Anti-PD-1(carrelizumab) + albumin paclitaxel + apatinib ± Fuzi Lizhong Pill	NA	30	ORR
Observational study								
2020-Wang L	ChiCTR2000032287	NSCLC	Bushen Tiaoyuan Recipe	Compound prescription	Anti-PD-1 + chemotherapy + Bushen Tiaoyuan Recipe	Anti-PD-1 + chemotherapy + placebo	55/55	ORR, AEs, and cancer fatigue score
2022-Zhang F	ChiCTR2200055330	Stomach Cancer	Wenyang Tongluo Recipe	Compound prescription	Anti-PD- 1(toripalimab) + Wenyang Tongluo Recipe + apatinib	Anti- PD-1(toripalimab) + apatinib	30/30	Tumor size and tumor marker

*TCM* traditional Chinese medicine, *RCT* randomized clinical trial, *ICIs* immune checkpoint inhibitors, *PFS* progression-free survival, *PD-1* programmed death-1, *NSCLC* none-small-cell lung cancer, *MST* median survival time, OS overall survival, *HCC* hepatocellular carcinoma, *PD-L1* programmed cell death-ligand 1, *AFP* alpha-fetoprotein, *DCR* disease control rate, *NA* not available, *LUSC* lung squamous cell carcinoma, *ORR* objective response rate, *AEs* adverse events

## TCM in the treatment and prevention of irAEs

The relatively high incidence of irAEs limits the use of ICIs. Effective precautions and treatments may help patients benefit more from ICI therapies. TCM for irAEs has been studied in a few experiments [100]. A practical reason is the lack of a robust preclinical mouse model; even though some models such as the ICI-associated myocarditis mouse model [101], are being developed.

Evidence from clinical studies and case reports suggests that TCM can exert therapeutic effects on irAEs (Table 3). A 56-year-old man with gastric carcinoma developed immune-related cystitis after five cycles of anti-PD-1 plus paclitaxel and tegafur treatment. The patients recovered without recurrence of their lower urinary tract symptoms after taking the TCM formulation Chai-Ling-Tang orally for 2 months [102]. Beyond compound formulation, Li et al. [103] reported that acupuncture treatment significantly relieved the symptoms of limb numbness and fatigue in a 63-year-old patient with immune-related Guillain-Barré syndrome (GBS). As demonstrated in this case, acupuncture effectively treated ICI-induced GBS in the absence of a significant response to intravenous gamma globulin.

**Table 3:** Clinical results of TCM in treatment and precaution of irAEs

Year-Author	Cancer type	TCM	Formulation	Immunotherapy	IrAE	Outcomes
RCT						
2022-Wu L [104]	NSCLC	Qigui Yishen Decoction	Compound prescription	Anti-PD-1 or anti-PD-L1	Immune checkpoint inhibitor-related AKI	Lower BUN, Scr, and higher eGFR
2022-Min M [105]	Malignant tumour	Heat-sensitive moxibustion	Moxibustion	Anti-PD-1	Prevention	Lower incidence of immune checkpoint inhibitor-related gastrointestinal toxicity
2021-Zhou Y [106]	Malignant tumour	Yifei Decoction	Yifei Decoction	ICIs	Immune checkpoint inhibitor-related pneumonitis	All grades reduced
2022-Xu Q [107]	Malignant tumour	Topical TCM	Compound prescription	ICIs	Immune checkpoint inhibitor-related skin toxicity	Lower incidence of immune checkpoint inhibitor-related skin toxicity
Case report						
2022-Wang Z [102]	gastric carcinoma	Chai-Ling -Tang	Compound prescription	Anti-PD-1(sintilimab) + paclitaxel + tegafur	Immune checkpoint inhibitor-related cystitis	Recovered
2022-Li J [103]	lung cancer	Acupuncture	Acupuncture	Anti-PD-1(tislelizumab) + docetaxel	Guillain–Barre Syndrome	Relieved

*TCM* traditional Chinese medicine, *irAE* immune checkpoint inhibitor-related adverse event, *NSCLC* none-small-cell lung cancer, *PD-1* programmed death-1, *PD-L1* programmed cell death-ligand 1, *AKI* acute kidney injury, *BUN* blood urea nitrogen, *Scr* serum creatinine, *eGFR* estimated glomerular filtration Rate, *ICIs* immune checkpoint inhibitors

The kidney function of 48 patients with acute kidney injury caused by ICIs was evaluated in a prospective randomized study [104]. Qigui Yishen Decoction significantly reduced blood urea nitrogen (BUN) (treatment group vs. control treatment:4.03 ± 0.82 mmol/L vs. 8.59 ± 1.08 mmol/L, *P* < 0.01), and serum creatinine (Scr) levels (treatment group vs. control treatment:60.03 ± 7.32 μmol/L vs. 150.59 ± 26.78 μmol/L, *P* < 0.01), and estimated glomerular filtration rate (eGFR) (treatment group vs. control treatment:60.03 ± 8.32 min/L vs. 47.59 ± 6.78 min/L, *P* < 0.05) when compared to corticosteroids alone. Meanwhile, the severity of acute kidney injury caused by ICIs has decreased. Similarly, another TCM compound formulation Yifei Decoction [106] in combination with corticosteroids had good efficiency in decreasing the grades of ICI-related pneumonia (combination group vs. corticosteroids group: 1.63 ± 0.74 vs. 1.82 ± 0.78, *P* < 0.05) and acceptable tolerability, but without statistical differences.

The external application of Chinese medicine seems to have attracted more attention in the field of irAEs (Table 4). Researchers have compared Pi-Yan-Ning, a Chinese patent medicine, to corticosteroids in the treatment of maculopapular rash caused by ICI grade 2–3 (ChiCTR2200059263). Additionally, electroacupuncture is being investigated as an effective treatment for irAE symptoms without affecting OS or progression-free survival (PFS) (ChiCTR2200059759).

**Table 4:** Ongoing clinical trials of TCM in treatment and precaution of irAEs

Year-author	Registry number	Study type	IrAE	TCM	Formulation	Intervention	Controlled	Sample size	Primary outcomes
2022-Ma X	ChiCTR2200059759	Cohort study	NA	Electroacupuncture	Acupuncture	Electroacupuncture stimulation of Zusanli acupoint	Without any acupuncture/Sham acupuncture stimulation of Zusanli acupoint	100/100/100	OS
2022-Shu Q	ChiCTR2200059263	RCT	Skin	Pi-Yan-Ning	Compound prescription	Pi-Yan-Ning	Corticosteroide	25/25	Median time of disease remission and drug efficacy

*IrAE* immune checkpoint inhibitors-related adverse event, *TCM* traditional Chinses medicine, *NA* not available, *OS* overall survival, *RCT* randomized controlled trial

TCM has also been regarded as having a preventive effect on irAEs in addition to its therapeutic benefit. According to Xu Q [107], Chinese medicine external treatment reduced the incidence of ICI-related cutaneous adverse events (treatment group vs. control group: 29.17% vs. 58.33%, *P* = 0.042). Heat-sensitization moxibustion as a traditional therapeutic technique [105] was tested in 40 patients who received anti-PD-1 therapy with a lower incidence of ICI-related gastrointestinal toxicity (treatment group vs. control group: 30.0% vs. 65.0%, *P* = 0.027) and grades (*P* = 0.007).

## Challenges and future perspectives

With the widespread use of ICIs, cancer patients with limited treatment have been living longer. However, the treatment is limited by primary and acquired resistance, unclear efficacy, and unpredictable toxicity. In China, oncology treatment has benefited from the integration of TCM and Western medicine for a long time. Multiple means of treatment can be applied to all stages of the tumor, focusing on different groups of the population based on syndrome differentiation [18]. TCM also helps improve the efficacy of ICIs and the prevention and treatment of irAEs, which is a completely new perspective. Therefore, research on the combination of TCM and ICIs is of practical significance.

The present studies, however, have many shortcomings. First, the bidirectional regulation of TCM combined with ICIs requires further research. Based on the literature review, TCM may have a synergistic effect and prevent irAEs owing to its multiple components, targets, and pathways. Astragalus, for example, can both enhance the efficacy of ICIs [92] and treat ICI-related acute kidney injury (AKI) [104]. Moxibustion improves immune function and lowers the incidence of ICI-related gastrointestinal side effects [105]. Despite this, research on the most obvious and crucial advantages of TCM combined with ICIs is underappreciated.

Second, our findings indicate some degree of incongruence between basic research and clinical trials. Basic research in this field is prone to utilizing TCM monomers rather than traditional compound prescriptions and Chinese medicine patents commonly utilized in clinical practice and clinical trials. In the case of Ginseng, which has been shown to have distinct synergic effects, has not been tested in clinical trials, let alone transformed into a mature medicine. Thus, in our review, we found that basic experiments and clinical studies focused on TCM in different lines, with little overlap. In the future, a stronger research team consisting of TCM physicians, Western medicine physicians, basic researchers, pharmacologists, and clinical trialists could collaborate more closely to improve translational research on TCM combined with ICIs. In addition, series studies are more important than single studies.

Third, it remains to be determined whether the existing standards for evaluating the clinical efficacy of modern medicine are applicable to TCM in combination with ICIs. In addition, we should pursue more diverse types of studies in this field, not just randomized clinical trials (RCTs) and case reports. The existing evaluation, RCT, only focuses on one medicine, and low-certainty case reports of the combination of TCM and ICIs cannot reflect its real-world effectiveness, even though the integration of TCM and Western medicine covers a large number of patients, particularly in China. The existing study modes and evaluation efficacy standards cannot adjust for TCM’s primary features of syndrome differentiation and treatment, individualized treatment, and holistic concepts. TCM combined with ICIs should be evaluated using new standards in future studies. Further clinical evidence, such as real-world studies, may reflect the real clinical condition of TCM in the era of immunotherapy.

Furthermore, there are new issues in immunotherapy that warrant TCM involvement. Examples include using TCM to overcome primary and acquired resistance to ICIs, examining the association between TCM syndromes, treatment regimens, the incidence of irAEs, combining TCM and ICIs for special populations, and artificial intelligence-assisted TCM combined with ICIs.

## Data Availability

Not applicable.

## Abbreviations

AKI: Acute kidney injury
AKT: Protein kinase B
BUN: Blood urea nitrogen
CCL5: Chemokine (C-C motif) ligand 5
CI: Confidence interval
CTLA-4: Cytotoxic T lymphocyte-associated protein 4
CRC: Colorectal cancer
COX2: Cyclooxygenase-2
CXCL9: Chemokine (C-X-C motif) ligand 9
DC: Dendritic cell
dLNs: Draining lymph nodes
eGFR: Estimated glomerular filtration rate
FasL: Factor related apoptosis ligand
GBS: Guillain-Barré syndrome
HCC: Hepatic cell carcinoma
HIF-1α: Hypoxia-inducible factor 1α
IDO: Indole-3-pyruvate
IL-2: Interleukin-2
ICIs: Immune checkpoint inhibitors
IFNγ: Interferon-γ
IrAEs: Immune checkpoint inhibitor-related adverse events
IRF1: Interferon regulatory factor 1
MDSCs: Myeloid-derived suppressor cells
mLNs: Mesenteric lymph nodes
M1: Macrophages 1
M2: Macrophages 2
NA: Not available
NKs: Natural killer cells
NSCLC: None-small-cell lung cancer
OR: Odds ratio
ORR: Objective response rate
OS: Overall survival
PD-1: Programmed death-1
PD-1int T cells: Intermediate PD-1 expression
PD-L1: Programmed cell death-ligand 1
PFS: Progression-free survival
PGE2: Prostaglandin E2
RCT: Randomized controlled trial
RR: Relative risk
SCFAs: Short chain fatty acids
Scr: Serum creatinine
TAMs: Tumor-associated macrophages
TCM: Traditional Chinese medicine
TGF-β: Tumor necrosis factor-β
TNF-α: Tumor necrosis factor-α
TME: Tumor microenvironment
Tregs: Regulatory cells
VEGF: Vascular endothelial growth factor
